# Anti-Ma2 Antibody-Associated Paraneoplastic Neurological Syndromes: A Pilot Study

**DOI:** 10.3390/brainsci11121577

**Published:** 2021-11-29

**Authors:** Yi Guo, Meng-Ting Cai, Qi-Lun Lai, Yang Zheng, Chun-Hong Shen, Yin-Xi Zhang

**Affiliations:** 1 Department of General Practice and International Medicine, Second Affiliated Hospital, School of Medicine, Zhejiang University, Hangzhou 310009, China; yiguo@zju.edu.cn; 2 Epilepsy Center, Second Affiliated Hospital, School of Medicine, Zhejiang University, Hangzhou 310009, China; 3 Department of Neurology, Second Affiliated Hospital, School of Medicine, Zhejiang University, Hangzhou 310009, China; mengtingc93@zju.edu.cn (M.-T.C.); yangzh92@zju.edu.cn (Y.Z.); shen_neurology@zju.edu.cn (C.-H.S.); 4 Department of Neurology, Affiliated Zhejiang Hospital, School of Medicine, Zhejiang University, Hangzhou 310013, China; laiqilun@zju.edu.cn

**Keywords:** anti-Ma2 antibody, paraneoplastic neurologic syndrome, sensorimotor neuropathy, multiple myeloma, factor analysis of mixed data

## Abstract

Paraneoplastic neurologic syndromes (PNSs) are a heterogeneous group of disorders caused by the remote effects of cancer with immune-mediated pathogenesis. Anti-Ma2 antibody was defined as one of the well-characterized onconeural antibodies that could help establish a definite PNS diagnosis. We aimed to report and explore patients with anti-Ma2 antibody-associated paraneoplastic neurologic syndrome (Ma2-PNS) who frequently exhibit sensorimotor neuropathy (SMN) using a new method of factor analysis of mixed data (FAMD). Clinical data from a case series of eight patients with definite diagnoses were retrospectively reviewed. FAMD conducted further analyses with a comprehensive visualization in R software. Our cohort, with a predominance of females (5/8), presented more frequently with SMN (4/8), followed by limbic encephalitis (LE) (3/8). Two patients with LE were found to have a testicular germ-cell tumor and a thymoma, respectively. In addition, a patient who developed chronic SMN was diagnosed with multiple myeloma (MM) involving multiple organs. FAMD exhibited the overall features into a two-dimensional coordinate and located each individual into their corresponding position with high relevance. It provided a clue for determining their potential relationships and predictors. Our findings indicated that Ma2-PNS could frequently involve the peripheral nervous system, MM might be one of its associated cancers with a presentation of chronic SMN, and FAMD might be a clinically valuable tool.

## 1. Introduction

Paraneoplastic neurologic syndromes (PNSs) are a heterogeneous group of disorders caused by the remote effects of cancer with immune-mediated pathogenesis [1,2]. They could affect any part of the nervous system from the cerebral cortex to neuromuscular junctions and muscle. Currently, the most widely used definite diagnostic criteria were firstly proposed by Graus et al. in 2004 as follows [3]: (1) a classical syndrome and cancer developing within 5 years of a neurological disorder’s diagnosis; (2) non-classical syndrome resolving or significantly improving after cancer treatment without concomitant immunotherapy, provided that the syndrome is not susceptible to spontaneous remission; (3) a non-classical syndrome with onconeural antibodies and cancer developing within 5 years of a neurological disorder’s diagnosis; and (4) a neurological syndrome (classical or not) with well-characterized onconeural antibodies and no cancer. Meanwhile, classical syndromes indicated the neurological syndromes associated frequently with cancer, including encephalomyelitis, limbic encephalitis (LE), subacute cerebellar degeneration, opsoclonus-myoclonus, subacute sensory neuronopathy, chronic gastrointestinal pseudo-obstruction, Lambert–Eaton myasthenic syndrome, and dermatomyositis.

Anti-Ma2 antibody was defined as one of the well-characterized onconeural antibodies that could help establish a definite PNS diagnosis [3]. Accordingly, anti-Ma2 antibody-associated PNS (Ma2-PNS) typically occurs in males with testicular germ-cell tumors and frequently presents with typical limbic, diencephalic, or brainstem dysfunction symptoms with abnormal MRI findings [4]. Ma2-PNS mainly involves the central nervous system, with fewer reports of peripheral syndromes. However, our study reported a cohort with predominantly non-classical syndromes and some unusual cancers [3,4,5,6]. Additionally, considering the complex and variable performance of this disease, a novel statistical method must explore the underlying relationships among each feature.

Factor analysis of mixed data (FAMD) is an exploratory method developed by the French school called Analyse des données and founded by Jean-Paul Benzécri [7,8]. It was initially used by Brigitte Escofier and Gilbert Saporta in 1979 [9,10], followed by Jérôme Pagès in 2002 [11]. It is a principal component method dedicated to analyzing a data set containing both quantitative and qualitative variables [7]. Compared with traditional statistical methods, FAMD is available for multivariate analyses with even a few individuals and qualitative variables concerning quantitative variables. Furthermore, it could help analyze similarities and their relationships after deconstructing the original complex data into fewer relevant factors [12,13]. It has been improved and applied in various fields, including describing the epidemiological characteristics of infectious diseases, such as COVID-19 and dengue vector mosquitoes; exploring and predicting the risk factors for health status, or diseases like rheumatoid arthritis and acute encephalitis; and monitoring environmental pollution [14,15,16,17,18,19,20]. As a result, we conducted FAMD to better visualize the correlations between each variable and individual and further explored the possible risk factors of outcomes.

## 2. Methods

### 2.1. Study Design

We retrospectively enrolled a cohort of 8 patients with definite Ma2-PNS [3] at the Second Affiliated Hospital School of Medicine Zhejiang University from January 2016 to December 2019. This study was approved by the ethics committee of Second Affiliated Hospital School of Medicine Zhejiang University (approval number: 2019-082). During the first hospitalization, data on demographic, clinical, paraclinical, and therapeutic characteristics were collected. The modified Rankin Scale (mRS) was employed to evaluate disease severity [21]. Prospectively collected follow-up data, including prognoses and further confirmation of associated cancers, were obtained from outpatient records or telephone calls every half year until death or May 2021.

Meanwhile, we recorded various laboratory data via serum/blood and cerebrospinal fluid (CSF) tests. In detail, white blood cells, protein, and IgG index (as determined by isoelectric focusing) in CSF corresponded to the immune-inflammatory response of the central nervous system, whereas tumor markers, coexisting antibodies, erythrocyte sedimentation rate (ESR), neutrophil-to-lymphocyte ratio (NLR), platelet-to-lymphocyte ratio (PLR), and lymphocyte to monocyte ratio (LMR) reflected peripheral or general conditions. Electroencephalogram (EEG), electromyography (EMG), magnetic resonance imaging (MRI), ^18^F-fluorodeoxyglucose positron emission tomography (FDG-PET), and computed tomography were performed to assess the individual pathological state and investigate the underlying cancers. The anti-Ma2 antibodies in serum and/or CSF were evaluated by commercial line blots (Euroimmun, Lübeck, Germany) and confirmed using indirect immunofluorescence tests on monkey cerebellum slides. Pathological examinations confirmed the presence of all cancers. 

### 2.2. Statistical Analysis

The descriptive analyses were conducted to describe these data by median (interquartile range [IQR]) for continuous data and number (percentage [%]) for categorical data. FAMD was used to summarize and visualize complex data and investigate their relationships. It combined principal component analysis functions for quantitative variables and multiple correspondence analysis for qualitative variables [7]. All data were normalized during the analysis to balance the influence of each set of variables. The variables comprised 12 quantitative and qualitative variables (Supplementary Materials Table S1). The analyses were conducted using R packages FactoMineR [22]. In our study, missing data were imputed using the Expectation-Maximization algorithm implemented in MissMDA package [23]. Corrplot package was used to display a correlation matrix [24]. A *p*-value of <0.05 was considered statistically significant.

## 3. Results

### 3.1. Clinical Features and Outcomes

Among 8 patients with a definite diagnosis, 5 were females (62.5%). The median age of onset was 56 years (IQR = 43.8–65.0 years). Except for 3 patients with a shorter disease course ranging from 19 days to 2 months, 5 patients had a chronic course lasting more than half a year. In 3 patients, limbic encephalitis was the only classical paraneoplastic neurological syndrome, whereas 4 patients experienced non-classical syndromes of sensorimotor neuropathies (SMN) and 1 patient exhibited motor neuron disease (MND). As a result, the proportion of central to peripheral syndromes was 3 to 5. Each of the 3 patients had multiple myeloma (MM) (IIIb), mixed germ cell tumor (testicular germ cell tumor and yolk sac tumor), and thymoma (IIIb). Three patients lost their lives during the follow-up while the rest remained unchanged or recovered to some degree. Among them, a 64-year-old female patient admitted to the hospital 2 years earlier for myasthenia gravis, and thymoma with lymphatic metastasis underwent thymoma resection and chemotherapy at that time. With presentation of LE at this attack, she progressed rapidly with coma and respiratory failure and was transferred to the intensive care unit following intubation. Unfortunately, she developed septic shock secondary to severe pulmonary infection, complicated with kidney failure, liver failure, and electrolyte disturbance. Finally, she lost her life as a result of cardiopulmonary arrest. Another 59-year-old female patient with chronic SMN achieved slight remission after treatment with intravenous methylprednisolone and intravenous immunoglobulin (IVIG). However, after a 19-month follow-up, she developed respiratory muscle paralysis and lost her life 1 month later. The last 47-year-old male patient with MND gradually developed dysphagia and neuromuscular respiratory failure even if treated with IVIG and eventually lost his life 7 months after the onset. Both of the latter two patients were cancer-free at the time of follow-up. Table 1 summarizes the details.

### 3.2. Paraclinical Findings

**Figure 1:**
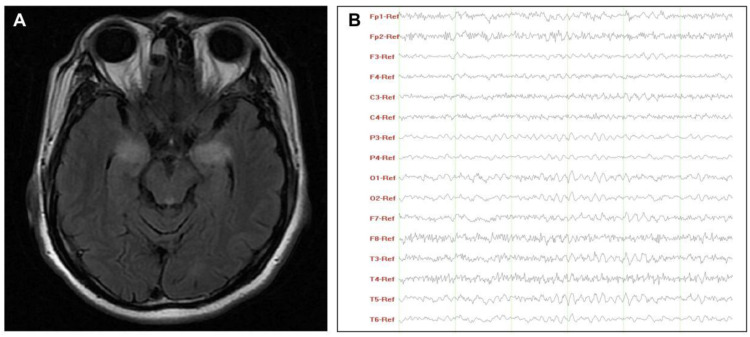
Characteristics of MRI (**A**) and EEG (**B**) of patient No.6. (**A**) T2-weighted images showed hyperintensity lesions on the bilateral mesial temporal lobe; (**B**) EEG revealed a widely diffuse distribution of theta waves with some low-amplitude fast activity. Abbreviations: EEG, electroencephalograms; MRI, magnetic resonance imaging.

According to paraclinical data (Table 2), two patients had Hashimoto’s thyroiditis and tested positive for autoimmune antibodies. The indicators associated with inflammation were increased in varying degrees. Only two patients (No. 2 and No. 7) had an increased ESR when the cutoff value was adjusted for age and sex (male: age in years/2; female: [age in years + 10]/2) [25]. As markers of systemic inflammatory response, NLR, LMR, and PLR values varied significantly, with ranges of 1.1–3.3, 84.5–255.8, and 2.1–6.8, respectively. Among CSF results of seven patients with lumbar puncture, one patient (No.6) with LE had significantly increased CSF cells, protein, and IgG index (>0.70) [26]. These findings indicated that the blood–brain barrier was damaged due to active inflammation, corresponding to his destructive lesions of bilateral mesial temporal lobe lesions in the MRI and slow-wave activity in the EEG (Figure 1).

### 3.3. FAMD

We performed FAMD as a comprehensive and global approach to identify similarities and differences between the characteristics of each individual and explore the potential prognostic clues. It could be explained as follows:

First, data variability was explained using five principal dimensions (Dims) in FAMD. The eigenvalues quantified the variation retained by each Dim and determined the number of Dims to consider. As illustrated in Supplementary Materials Table S1, the first Dim had larger eigenvalues than subsequent Dims, corresponding to the directions with the maximum variation in the data set. To establish a coordinate, we selected the first 2 Dims with a total of 45.1% variations, as Dim 1 and 2 each accounted for 24.6% and 20.5%, respectively.

**Figure 2:**
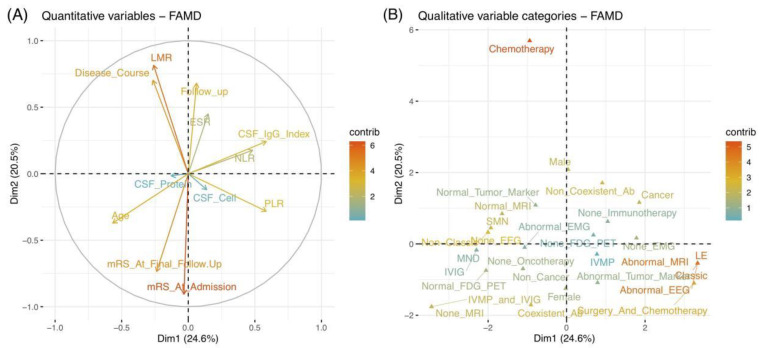
Correlation plots of quantitative (**A**) and qualitative (**B**) variables. The X-axis and Y-axis represent Dim 1 (24.6%) and Dim 2 (20.5%) with respective cumulative variance percent in brackets. The variables were highlighted by their contribution values with arrows (**A**) and points (**B**). A higher value with a redder color indicates better contributions in Dims. (**A**) The correlation circle indicates that the closer the arrow to the axis represented greater contribution of the variable on that axis (Dim). The angle of the 2 variables suggests their positive or negative correlation. (**B**) The scatter plot reveals the contributions of each variable in the different poles of Dims. A good representation of a variable was reflected by the farther distance between the point of the variable and the origin on the factor map. Abbreviations: ab, antibody; CSF, cerebrospinal fluid; Dim, dimension; EEG, electroencephalograms; EMG, electromyography; ESR, erythrocyte sedimentation rate; FAMD, factor analysis of mixed data; FDG-PET, ^18^F-fluorodeoxyglucose positron emission tomography; IVMP, intravenous methylprednisolone; IVIG, intravenous immunoglobulin; LE, Limbic encephalitis; LMR, lymphocyte to monocyte ratio; MND, motor neuron diseases; MRI, magnetic resonance imaging; mRS, modified Rankin Scale; NLR, neutrophil-to-lymphocyte ratio; PLR, platelet-to-lymphocyte ratio; SMN, sensorimotor neuropathies.

**Figure 3:**
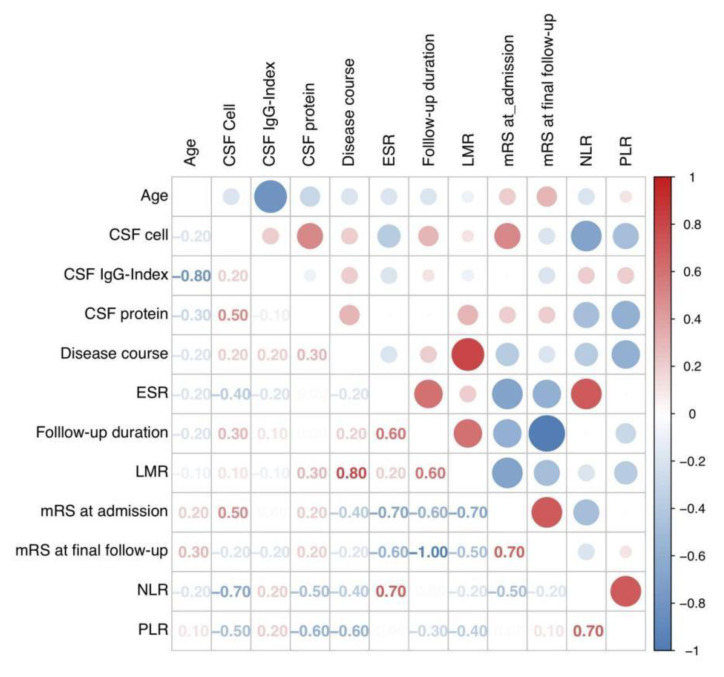
Correlation matrix plot showing Pearson’s correlation coefficient between each variable. Abbreviations: CSF, cerebrospinal fluid; ESR, erythrocyte sedimentation rate; LMR, lymphocyte-to-monocyte ratio; mRS, modified Rankin Scale; NLR, neutrophil-to-lymphocyte ratio; PLR, platelet-to-lymphocyte ratio.

We then analyzed a correlation between variables and the main two Dims using three parameters: coordinates, squared cosine (cos2), and contribution (Supplementary Materials Table S2). Correlation plots were employed to visualize the major variables associated with each Dim (Supplementary Materials Figure S1). Among them, the parameter of the coordinate of variables was used to create a scatter plot. The cos2 indicated the quality of representation for variables on the factor map, as indicated by the angle between the variable point and the axis. In addition, the contribution was determined using the distance of the perpendicularly project point of the variable on the corresponding Dim axis. Each variable is positioned in different quadrants of the two Dims, corresponding to their positive or negative contributions. Several variables were highlighted in a redder color, such as “LMR” and “mRS At Admission”, in the quantitative plot (Figure 2A), as well as “Oncotherapy”, “Abnormal MRI”, and “Abnormal EEG” in the qualitative plot (Figure 2B). Figure 3 supplements the bar plots for contributions. As a result, the mainly correlated variables to Dim 1 were “MRI”, “Syndrome”, “Classic types”, and “EEG” variables, whereas “mRS At Admission”, “Oncotherapy”, and “LMR” were more representative for Dim 2. We further explored the relationships between each variable in two steps. Above all, we separately analyzed the quantitative and qualitative variables for detailed observations. In addition, the overall analysis of the two data sets was performed subsequently for comprehensive visualization.

The relationships between quantitative and qualitative variables were separately explored. As a result, the correlations between each two quantitative variables were indicated by their angle in the correlation circle (Figure 2A). An acute and obtuse angle respectively suggested positive and negative correlations, while the orthogonal angle indicated a limited correlation. After the quantitative analysis in Figure 3, the variables of “LMR” and “Disease course” demonstrated a good positive correlation (r = 0.80, *p* = 0.019), followed by the correlation between “PLR” and “NLR”, “mRS at final follow-up”, and “mRS at admission” (r = 0.70, *p* = 0.058; r = 0.70, *p* = 0.067). In contrast, negative correlations were obvious between “mRS at final follow-up” and “follow-up duration” (r = −1.00, *p* = 0.014), as well as between “age” and “CSF IgG index” (r = −0.80, *p* < 0.001). It could be summarized that the final outcomes of patients scored by mRS might be linked to the follow-up duration and initial severity, and patients with mild onset might have better short-term outcomes. The laboratory results revealed complicated relationships with each other, indicating their potential co-action to pathogenic mechanisms.

**Figure 4:**
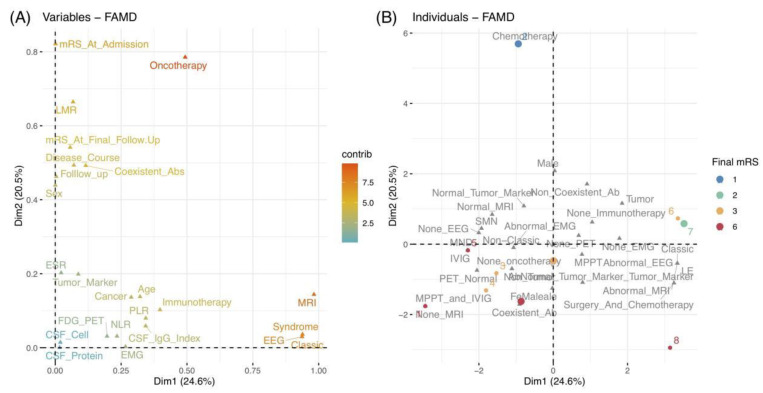
(**A**) Correlations between quantitative and qualitative variables in the first quadrant of 2 Dims by the value of the contributions. (**B**) Plot of 8 individuals colored by their mRS scores at the final follow-up. Abbreviations: ab, antibody; CSF, cerebrospinal fluid; Dim, dimension; EEG, electroencephalograms; EMG, electromyography; ESR, erythrocyte sedimentation rate; FAMD, factor analysis of mixed data; FDG-PET, ^18^F-fluorodeoxyglucose positron emission tomography; IVMP, intravenous methylprednisolone; IVIG, intravenous immunoglobulin; LE, Limbic encephalitis; LMR, lymphocyte-to-monocyte ratio; MND, motor neuron diseases; MRI, magnetic resonance imaging; mRS, modified Rankin Scale; NLR, neutrophil-to-lymphocyte ratio; PLR, platelet-to-lymphocyte ratio; SMN, sensorimotor neuropathies.

Referring to qualitative variables, their proximity on the plot indicated similar characteristics (Figure 2B). In addition, their relationships could be reflected by their distributions, corresponding to quantitative variables. Figure 4A depicts a global pattern within the data in the first quadrants. This means that variables distant from the origin continued to contribute significantly to Dims, and those in close proximity could be explained to each other. For instance, the close position in the variables of “EEG”, “MRI”, “syndrome”, and “classic” suggested their similar profiles, e.g., patients with classic syndrome of LE usually had abnormal EEG and MRI.

**Figure 5:**
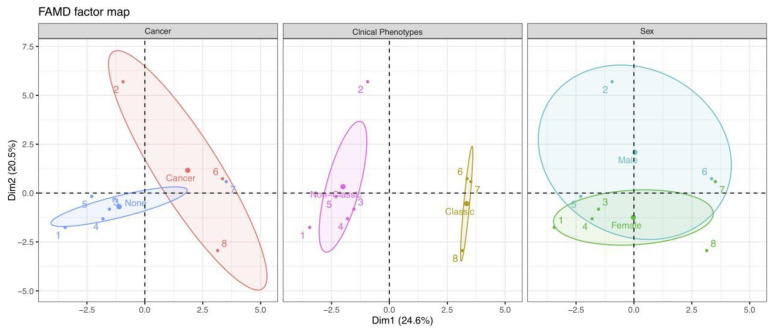
Individual plots corresponding to the classification features of cancer, clinical phenotypes, and sex. Each color represents individuals in a specific feature, and the confidence ellipses indicate their potential relationships. Abbreviation: Dim, dimension.

Finally, we could examine the similarities and differences between individual profiles. As depicted in Figure 4B, different colored points indicate patients with corresponding mRS scores at the final follow-up. As previously stated, the positions of individuals on these two Dims can reflect their characteristics. Therefore, as an example, we could speculate that patient 2 close to Dim 2 underwent chemotherapy (combined with cancer) and eventually achieved a good outcome with an mRS of 1. Meanwhile, patient 2 might have mild severity at onset, a higher LMR value, and longer follow-up duration, based on the quantitative variables of mRS at admission, LMR, and the follow-up duration, which contributed significantly to Dim 2. Moreover, we could divide and color each individual by other variables and explore their relationships using the ellipses of the confidence intervals (Figure 5).

## 4. Discussion

We reported a series of eight patients with Ma2-PNS and analyzed their data using a novel method called FAMD. Interestingly, our cohort demonstrated a predominance of peripheral syndromes associated with SMN and identified the first Ma2-PNS associated with MM. The use of FAMD led to a comprehensive visualization of each individual and their numerous associating variables.

According to previous descriptions about demographic, clinical, and prognostic features of Ma2-PNS, typical presentations included central nervous system syndromes, such as limbic, diencephalic, or brainstem dysfunction, with abnormal MRI findings [4,5,27,28,29]. Younger males with potential testicular germ cell tumors usually responded well to treatment, and had stable neurological syndromes and long-term survival. However, isolated peripheral involvement was reported infrequently, including two cases of radiculopathy and two cases of multiple mononeuropathies [28,30]. Our novel finding of predominant presentation of SMN could be explained by the pathological results. The paraneoplastic antigen Ma2 recognized by anti-Ma2 antibodies was expressed in central and peripheral neurons, including the brain and spinal cord, dorsal root ganglia, intestinal autonomic neurons, adrenal medullary ganglion cells, and cancers [6,27,28,29]. In general, as a rare disease, its clinical features should be further evaluated using larger sample sizes. Additional in-depth mechanism studies should be conducted to explore the difference between central and peripheral involvement. In addition, our patients had high mortality even after oncotherapies and immunosuppressive treatments. Given that anti-Ma antibodies were directed against intracellular antigens, immunotherapies might be useless [28,31], even though an earlier treatment (within the first month after onset) was suggested as a possible way to avoid irreversible neuronal damage.

A novel cancer of MM was discovered in a 53-year-old female with mild chronic SMN. Although multiple organs were involved in MM due to plasma cell infiltration or immunoglobulin deposition, neurologic manifestations were uncommon [32]. Apart from radiculopathy, peripheral neuropathy was a rare complication in MM, usually caused by associated immunoglobulin light-chain (AL) amyloidosis or polyneuropathy, organomegaly, endocrinopathy, M-protein, skin changes (POEMS) syndrome [30]. As the former consisted of 20–40% of patients in MM, about 25% of them had painful and sensory or sensory-motor symptoms, along with carpal tunnel syndrome, macroglossia, purpura, nephrotic syndrome, congestive heart failure, and orthostatic hypotension [33,34,35]. However, this diagnosis should be supported by positive amyloid staining with Congo red in related tissue or by the presence of amyloid fibrils on electron microscopy [36]. Meanwhile, the presentations of our patient were inconsistent with the criterion of POEMS syndrome. Moreover, MM was reported as being associated with cancer in Stiff person syndrome with positive anti-glutamic acid decarboxylase antibodies [37]. In addition, a combination of diffuse large B cell lymphoma and smoldering MM with anti-ZIC4 antibody was reported in a patient with indolent paraneoplastic cerebellar degeneration [38]. However, no combination of MM and anti-Ma2 antibodies was reported. Unfortunately, because our patient did not complete antigen expression and tissue biopsy for the diagnosis of Ma2-PNS and AL amyloidosis, respectively, her definitive diagnosis remained unknown. Above all, potential relationships between these two conditions should be considered and further validated.

We proposed a novel way of analyzing the clinical performance of eight patients using statistical techniques that enable visualization through the use of figures and tables. Although PNS is a rare disease, it exhibited complex and diverse features that could mainly be descriptive generalization rather than further systematic analyses. As the first application in observing and analyzing PNS characteristics, FAMD provided clues of similarities and differences between complicated features and indicated risk factors for outcomes via a coordinate. This means that we could speculate other potentially related characteristics and even possible prognoses in certain clinical features of a patient. However, this was only the first step in establishing their correlations; additional details should be validated with more evidence. Meanwhile, our study discovered a novel utility of NLR, LMR, and PLR in PNS. Previous research indicated their diagnostic, prognostic, and predictive roles in multiple diseases, such as cancers, coronary heart disease, pancreatitis, inflammatory autoimmune diseases, immune therapies, traumas, and mood disorders [39,40,41,42]. Our study found their value to reflect the two Dims of each individual, such as disease onset age, disease course, and disease severity. These inflammatory markers might be used to monitor the severity of the inflammatory response and identify deeper pathogenic mechanisms. Above all, this visualization tool might be beneficial for future prediction of prognosis and disease management.

Several limitations should be underlined in our study. First, since this single-center retrospective study based on 2004 PNS criteria included only eight patients, with the advent of the recently proposed 2021 diagnostic criteria [43], larger samples worldwide using new criteria should be collected for a better exhibition. In addition, a longer follow-up should be performed to track the occurrence of potential cancers and their subsequent prognosis. Second, we should acknowledge that antibody test results might reveal some false positives even if we used two methods, which could affect diagnostic accuracy. Moreover, while we focused on a few specific variables correlated with FAMD, other potential correlated factors might be considered and cannot be ruled out. Consequently, prospective studies with larger cohorts with more diverse syndromes are required to explore more details of Ma2-PNS and validate the efficacy of FAMD and its clinical utility.

## 5. Conclusions

Ma2-PNS could frequently involve the peripheral nervous system with a presentation of SMN. MM might be a potential associated cancer. All in all, Ma2-PNS has poor prognosis. The new application of FAMD might be beneficial in identifying distinct clinical profiles. It could prompt further reflection on the relationships between individual clinical features. To go further, in light of our results, it will be meaningful to target specific therapeutic strategies for those most at risk.

## Figures and Tables

**Table 1:** Clinical characteristics and outcomes of 8 patients with anti-Ma2 antibody-associated paraneoplastic neurologic syndromes.

No	Sex	Age at Onset, y	Clinical Symptoms	Syndromes	Associated Cancer/Diagnostic Time	Oncotherapy	Immunotherapy	Follow-Up Duration, m	mRs Score on Admission/at Final Follow-Up
1	F	59	Limb numbness and weakness	Chronic SMN	-	-	IVMP + IVIG	20.0	4/6
2	M	53	Limb numbness and pain	Chronic SMN	Multiple myeloma (IIIb)/16 m after onset	Chemotherapy	None	57.5	1/1
3	F	69	Limb numbness and weakness	Chronic SMN	-	-	None	41.1	4/3
4	F	68	Limb numbness and weakness	Subacute SMN	-	-	IVMP	44.5	4/3
5	M	47	Muscle weakness and atrophy	MND	-	-	IVIG	7.0	4/6
6	M	34	Drowsy, memory loss, hallucinations	LE	Mixed germ cell tumors */2 m after onset	Surgery + chemotherapy	IVMP	42.8	4/3
7	F	29	Psychiatric symptoms	LE	-	-	None	46.8	3/2
8	F	64	Memory loss	LE	Thymoma (IIIb) */2 y before onset	Surgery + chemotherapy	None	0.0	4/6

Abbreviations: F, female; IVMP, intravenous methylprednisolone; IVIG, intravenous immunoglobulin; LE, Limbic encephalitis; M, male; m, month; MND, motor neuron diseases; mRs, modified Rankin Scale; SMN, sensorimotor neuropathies; y, year; -, negative result or not applicable. * Confirmed by pathological examination.

**Table 2:** Laboratory, electrophysiology, and imaging data of eight patients with antibody-associated paraneoplastic neurologic syndromes at the first attack.

No	Tumor Marker	Coexistent Antibodies	ESR, mm/h	NLR	PLR	LMR	CSF			MRI	FDG-PET	EEG	EMG
							Cells/mL	Protein, mg/dL	IgG Index				
1	Normal	Anti-ATG, anti-TPO, anti-TG	21.0	1.5	84.9	3.9	3	103	0.11	-	-	-	Demyelinating
2	Normal	Monoclonal protein, IgG, Kappa light	30.0 ^	2.6	125.0	6.8	0	43.3	0.48	Normal	-	-	Demyelinating
3	CA125	Anti-β2GPI	19.0	1.4	93.0	2.9	6	43.5	0.31	Normal	-	-	-
4 *	β-hCG	Anti-ATG	14.0	1.8	163.1	3.8	4	31.5	0.43	Normal	Normal	-	Demyelinating and axonal
5	Normal	Normal	4.0	1.6	120.7	3.3	-	-	-	Normal	Normal	-	Denervation of multiple muscles in cervical, thoracic, and lumbosacral regions
6 *	AFP, PSA	Normal	9.0	1.1	115.1	4.8	10	87.8	0.90	BMTL	-	Slow wave	-
7	Normal	Normal	33.0 ^	3.3	160.4	2.3	2	47.6	-	BMTL	-	Slow wave	-
8	CEA, CA211, SCC *	Anti-ATG, anti-TPO	15.0	2.9	225.8	2.1	0	27.5	-	BMTL	-	Slow wave	Decremental response on slow RNS

Abbreviations: AFP, alpha fetoprotein; ATG, anti-thyroglobulin abs; BMTL, bilateral mesial temporal lobe lesions; β2GPI, beta-2 glycoprotein 1; CA, carbohydrate antigen; CAMP, compound motor action potential; CEA, carcinoembryonic antigen; CSF, cerebrospinal fluid; EEG, electroencephalograms; EMG, electromyography; ESR, erythrocyte sedimentation rate; FDG-PET, ^18^F-fluorodeoxyglucose positron emission tomography; hCG, human chorionic gonadotrophin; LMR, lymphocyte-to-monocyte ratio; MRI, magnetic resonance imaging; NLR, neutrophil-to-lymphocyte ratio; PLR, platelet-to-lymphocyte ratio; PSA, prostate-specific antigen; RNS, repetitive nerve stimulation; SCC, squamous cell carcinoma antigen; TG, thyroglobulin; TPO, thyroperoxidase; -, not done or no date available, * positive anti-Ma2 abs in CSF, while others were serum positive; ^ increased ESR after adjusting for age and sex.

## Data Availability

Anonymized data not published within this article will be made available upon reasonable request from any qualified investigator within 5 years after publication.

## Supplementary Materials

The following are available online at https://www.mdpi.com/article/10.3390/brainsci11121577/s1, Figure S1: Contributions of variables in Dims 1 and 2, Table S1: Eigenvalue and variability explained by the FAMD., Table S2: Coordinates, cos2 and contributions of the variables with the first 2 dimensions of FAMD.
