# Supplementary material for: Anti-Ma2 Antibody-Associated Paraneoplastic Neurological Syndromes: A Pilot Study
Source: Brain Sci. 2021 Nov 29;11(12):1577. doi: 10.3390/brainsci11121577 (PMC8699657; doi:10.3390/brainsci11121577)
Supplement: Supplementary file 1 [file brainsci-11-01577-s001.zip › brainsci-1433704-supplementary.pdf]

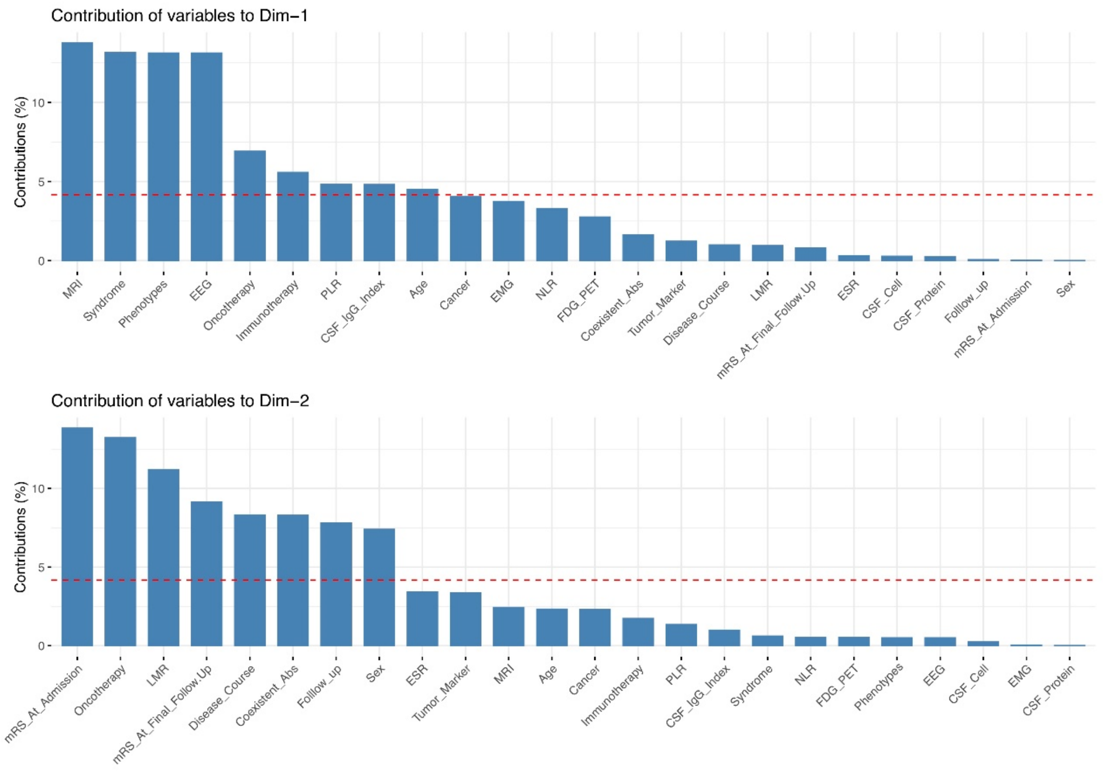

Figure S1. Contributions of variables in Dims 1 and 2.

The red dashed line on the graph above indicates the expected average contribution. If the contribution of variables were uniform, the expected value would be 1/length (variables) = 1/10 = 10%. For a given Dim, a variable with a contribution larger than this cutoff could be considered important in contributing to Dim.

Abbreviations: ab, antibody; CSF, cerebrospinal fluid; Dim, dimension; EEG, electroencephalograms; EMG, electromyography; ESR, erythrocyte sedimentation rate; FAMD, factor analysis of mixed data; FDG-PET, ^{18}F-fluorodeoxyglucose positron emission tomography; IVMP, intravenous methylprednisolone; IVIG, intravenous immunogloblin; LE, Limbic encephalitis; LMR, lymphocyte to monocyte ratio; MND, motor neuron diseases; MRI, magnetic resonance imaging; mRS, modified Rankin Scale; NLR, neutrophil-to-lymphocyte ratio; PLR,

platelet-to-lymphocyte ratio; SMN, sensorimotor neuropathies.

Table S1. Eigenvalue and variability explained by the FAMD.

	Eigenvalue variance	Percentage of variance	Cumulative variance percent
Dim.1	7.140	24.620	24.620
Dim.2	5.937	20.471	45.090
Dim.3	4.949	17.067	62.158
Dim.4	4.076	14.055	76.213
Dim.5	3.177	10.955	87.168

Abbreviation: Dim, dimension.

Table S2. Coordinates, cos2 and contributions of the variables with the first 2 dimensions of FAMD.

	Coordinates		Cos2 / quality of representation		Contributions	
	Dim.1	Dim.2	Dim.1	Dim.2	Dim.1	Dim.2
Age	0.3217	0.1379	0.1035	0.0190	4.5063	2.3228
Disease course	0.0705	0.4934	0.0050	0.2435	0.9869	8.3114

Follow-up duration	0.0039	0.4627	0.0000	0.2141	0.0547	7.7942
NLR	0.2343	0.0312	0.0549	0.0010	3.2812	0.5248
PLR	0.3436	0.0798	0.1180	0.0064	4.8120	1.3439
LMR	0.0677	0.6645	0.0046	0.4415	0.9478	11.1930
ESR	0.0222	0.2024	0.0005	0.0410	0.3113	3.4088
CSF Cell	0.0193	0.0145	0.0004	0.0002	0.2696	0.2438
CSF Protein	0.0166	0.0003	0.0003	0.0000	0.2324	0.0045
CSF IgG Index	0.3432	0.0587	0.1178	0.0034	4.8069	0.9887
mRS at admission	0.0011	0.8215	0.0000	0.6749	0.0149	13.8388
mRS at final follow-up	0.0565	0.5416	0.0032	0.2934	0.7918	9.1240
Sex	0.0001	0.4394	0.0000	0.1930	0.0018	7.4009
Syndrome	0.9385	0.0362	0.4404	0.0007	13.1452	0.6094
Clinical phenotypes	0.9366	0.0296	0.8773	0.0009	13.1188	0.4993

Cancers	0.2883	0.1365	0.0831	0.0186	4.0381	2.2990
Tumor marker	0.0874	0.1988	0.0076	0.0395	1.2244	3.3490
Coexistent abs	0.1157	0.4924	0.0134	0.2424	1.6207	8.2938
MRI	0.9819	0.1440	0.4821	0.0104	13.7533	2.4252
EMG	0.2666	0.0027	0.0711	0.0000	3.7342	0.0461
EEG	0.9366	0.0296	0.8773	0.0009	13.1188	0.4993
FDG-PET	0.1966	0.0310	0.0387	0.0010	2.7537	0.5228
Immunotherapy	0.3976	0.1026	0.0527	0.0035	5.5684	1.7278
Oncotherapy	0.4931	0.7853	0.1216	0.3084	6.9067	13.2288

Abbreviations: abs, antibodies; cos2, squared cosine; CSF, cerebrospinal fluid; Dim, dimension; EEG, electroencephalograms; EMG, electromyography; ESR, erythrocyte sedimentation rate; FAMD, factor analysis of mixed data; FDG-PET, ^{18}F-fluorodeoxyglucose positron emission tomography; LMR, lymphocyte to monocyte ratio; MRI, magnetic resonance imaging; mRs, modified Rankin Scale; NLR, neutrophil-to-lymphocyte ratio; PLR, platelet-to-lymphocyte ratio.
